# *Orf-I* and *Orf-II*-Encoded Proteins in HTLV-1 Infection and Persistence

**DOI:** 10.3390/v3060861

**Published:** 2011-06-17

**Authors:** Dustin Edwards, Claudio Fenizia, Heather Gold, Maria Fernanda de Castro-Amarante, Cody Buchmann, Cynthia A. Pise-Masison, Genoveffa Franchini

**Affiliations:** Animal Models and Retroviral Vaccines Section, Center for Cancer Research, National Cancer Institute, National Institutes of Health, Bethesda, Maryland, MD 20892, USA; E-Mails: edwardsd2@mail.nih.gov (D.E.); feniziac@mail.nih.gov (C.F.); goldhl@mail.nih.gov (H.G.); amarantem@mail.nih.gov (M.F.d.C.-A.); buchmannc@mail.nih.gov (C.B.); masisonc@mail.nih.gov (C.A.P.-M.)

**Keywords:** human T-cell leukemia/lymphoma virus type-1, HTLV-1, ORF-I, ORF-II, p8, p12, p13, p30

## Abstract

The 3′ end of the human T-cell leukemia/lymphoma virus type-1 (HTLV-1) genome contains four overlapping open reading frames (ORF) that encode regulatory proteins. Here, we review current knowledge of HTLV-1 *orf-I* and *orf-II* protein products. Singly spliced mRNA from *orf-I* encodes p12, which can be proteolytically cleaved to generate p8, while differential splicing of mRNA from *orf-II* results in production of p13 and p30. These proteins have been demonstrated to modulate transcription, apoptosis, host cell activation and proliferation, virus infectivity and transmission, and host immune responses. Though these proteins are not essential for virus replication *in vitro*, p8, p12, p13, and p30 have an important role in the establishment and maintenance of HTLV-1 infection *in vivo*.

## 1. Introduction

Human T-cell leukemia/lymphoma virus type-1 (HTLV-1) is an oncogenic retrovirus first discovered in 1980 in T-cells of a patient with cutaneous T-cell lymphoma [1,2]. HTLV-1 is the etiological agent of two major diseases: adult T-cell leukemia (ATL), a disease characterized by malignant proliferation of CD4^+^ T-lymphocytes, and tropical spastic paraparesis/HTLV-1-associated myelopathy (TSP/HAM), a neurodegenerative condition [3,4]. HTLV-1 is also associated with other clinical disorders including HTLV-1-associated arthropathy, HTLV-1-associated uveitis, infective dermatitis, and polymyositis [5,6]. HTLV-1 primarily infects CD4^+^ T-cells and has been detected in *ex vivo* CD8^+^ T-cells, dendritic cells (DC), and B-cells from infected individuals. While cell-free virions have been shown to efficiently infect DCs *in vitro*, HTLV-1 is believed to be transmitted to T-cells and DCs mostly by cell-to-cell contact through a virological synapse, biofilm-like extracellular viral assemblies, or cellular conduits [7–10]. An estimated 10–20 million people worldwide are infected with HTLV-1 [11]. While the majority of HTLV-1-infected individuals remain asymptomatic, a low percentage of patients develop either ATL (3–5%) or TSP/HAM (0.3–2%) after a long period of clinical latency [12–19].

**Figure 1.**
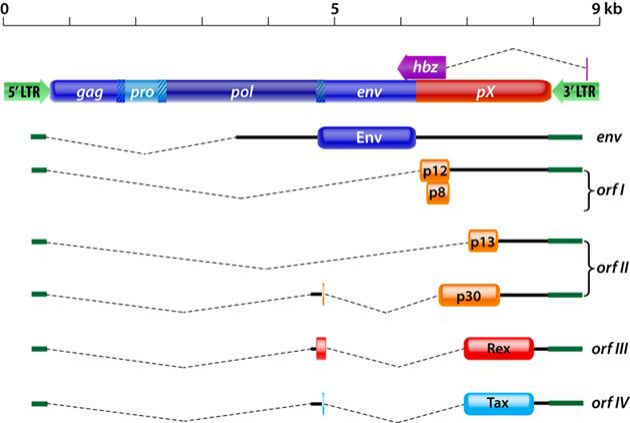
A scheme of the human T-cell leukemia/lymphoma virus type-1 (HTLV-1) genome. Spliced mRNAs and encoded proteins for *orf-I* and *orf-II* are shown. *Orf-I* encodes for the p12 protein which can be proteolytically cleaved at the amino terminus to generate the p8 protein. The p30 protein is translated from doubly spliced mRNA transcribed from *orf-II* and the 5′ end of *env*. The p13 protein is translated from singly spliced mRNA transcribed from *orf-II* and corresponds to the carboxyl terminus of p30.

As shown in Figure 1, the HTLV-1 genome contains the typical retroviral structural and enzymatic genes *gag*, *pro*, *pol*, and *env* [13]. In addition, a region located between *env* and the 3′ long terminal repeat (LTR), contains four partially overlapping open reading frames (ORF) [13]. This unique region encodes several regulatory proteins through the use of alternative splicing and internal initiation codons [20–22]. *Orf-I* produces the p12 protein which can be proteolytically cleaved at the amino terminus to generate the p8 protein, while differential splicing of mRNA from *orf-II* results in production of the p13 and p30 proteins [20–23]. *Orf-III* and *orf-IV* encode for the Rex and Tax proteins, respectively, and an antisense mRNA transcribed from the 3′ LTR that generates the HTLV-1 basic leucine zipper (HBZ) protein [24–26].

Tax and Rex are required for viral replication. Tax is a potent transcriptional transactivator of viral gene expression. Tax also regulates the expression of several cellular genes, including those involved in cell proliferation, cell cycle progression, apoptosis, and DNA damage responses. Rex is a post-transcriptional regulator that facilitates nuclear export of unspliced and singly spliced viral mRNA. In addition, Rex inhibits splicing and transport of doubly spliced mRNA. HBZ is a negative regulator of Tax-mediated transactivation and thus suppresses viral expression. For further detailed information about Rex, Tax, and HBZ, the reader is referred to recent reviews [27–30]. In this review, we will focus on the current knowledge of the functions of the proteins encoded by *orf-I* and *orf-II*: p8, p12, p13, and p30.

In contrast to Tax and Rex, *orf-I* and *orf-II* are dispensable for viral replication *in vitro* yet are important for viral persistence *in vivo* [31]. Early work demonstrated that in the rabbit model, *orf-I* was required for viral infectivity while *orf-II* was required to maintain high viral load [32,33]. Further work in the rabbit model showed reversion of HTLV-1 clones lacking p30 to the wildtype p30-expressing virus, suggesting the importance of p30 to HTLV-1 viral persistence [34]. However, in these early studies the HTLV-1 clones that were used contained a frameshift that affected *hbz*, making it unclear as to whether these effects were due to the loss of *hbz* or *orf-I* and *orf-II*-encoded proteins. In a more recent study, the ablation of p12/p8, p30, or HBZ impaired the establishment of persistent infection in the macaque model [35]. Nevertheless, ablation of these proteins did not affect viral replication in the rabbit model [35]. The *orf-I* and *orf-II*-encoded proteins are able to modulate a diverse range of viral and cellular mechanisms including transcriptional regulation, mitochondrial function, cell cycle progression, host cell activation and proliferation, apoptosis, virus infectivity and transmission, and host immune responses. Though these proteins are not essential for virus replication *in vitro*, p8, 12, p13, and p30 have an important role in the establishment and maintenance of HTLV-1 infection *in vivo*.

## 2. HTLV-1 p12 and p8

HTLV-1 *orf-I* encodes the 99 amino acid p12 protein which can be proteolytically cleaved at the amino terminus to generate the p8 protein (Figure 1). Computational analysis of the amino acid sequence of p12 predict the existence of a noncanonical endoplasmic reticulum (ER) retention/retrieval signal between amino acids 1–5, two putative leucine zipper (LZ) motifs, two putative transmembrane domains between amino acids 12–30 and amino acids 48–67, a calcineurin-binding motif between amino acids 70–86, four putative proline-rich (PXXP) Src homology 3 (SH3)-binding domains, and a putative adaptin motif [23,36]. These structural features may contribute to protein localization, homodimerization, and protein-protein interactions. The p12 protein exhibits amino acid similarity with a portion of the bovine papillomavirus (BPV)-transforming E5 protein, except that E5 does not carry putative SH3 binding motifs [37,38]. The p12 protein undergoes complex post-translational modifications through proteolytic cleavage. The first cleavage occurs between amino acid positions 9 and 10 and is followed by a second cleavage between amino acids 29 and 30 [23]. The first proteolytic cleavage removes the ER retention/retrieval signal at the amino terminus of p12, while the second cleavage generates the p8 protein [23]. The p12 protein localizes to cellular endomembranes, particularly within the ER and Golgi apparatus, while p8 traffics to lipid rafts at the cell surface and is recruited to the immunological synapse upon T-cell receptor (TCR) ligation [23,39–41].

The singly spliced mRNA encoding p12/p8 has been detected *in vitro* and in *ex vivo* HTLV-1-infected T-cells and macrophages [42]. The p12 recombinant protein is recognized in serum from humans infected with HTLV-1 and rabbits experimentally infected with HTLV-1 [43]. In addition, a cytotoxic T-lymphocyte (CTL) response to *orf-I* products can be detected in HTLV-1-infected individuals [44]. Two natural variants of the p12 protein have been identified; one variant carries a lysine residue at position 88 and is commonly found in HTLV-1 strains from TSP/HAM patients while the second variant carries an arginine residue at position 88 and is found in HTLV-1 strains from all ATL patients and healthy carriers studied. The R88 variant protein has a much greater stability compared to the K88 variant, which is ubiquitinated and rapidly degraded by the proteasome [45].

### 2.1. T-Cell Signaling

#### 2.1.1. Calcium Release

**Figure 2.**
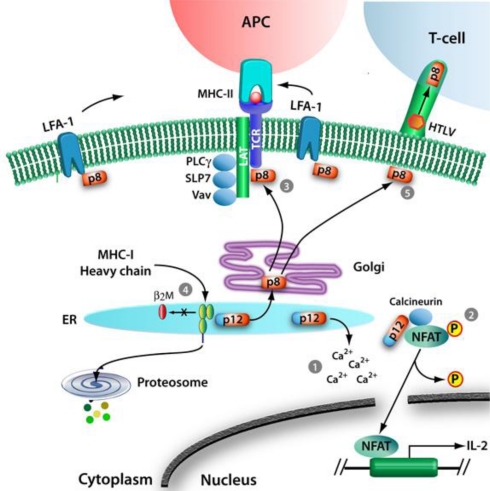
Functions of p12 and p8. In the ER, p12 is proteolytically cleaved at the amino terminus to generate p8, which traffics to the cell surface through the secretory pathway. (**1**) In the ER, p12 mediates Ca^2+^ release, which enables (**2**) either calcineurin binding of NFAT and subsequent dephosphorylation, nuclear translocation, and upregulation of the IL-2 gene, or p12 binding to calcineurin and inhibition of NFAT activation. (**3**) Upon trafficking through the secretory pathway, p8 localizes at the immunological synapse where it interacts with LAT and inhibits proximal TCR signaling. (**4**) In the ER, p12 binds the immature heavy chains of the MHC-I and prevents their interactions with the β_2_-microglobulin, leading MHC-I degradation by the proteosome. (**5**) At the cell surface, p8 increases the clustering of LFA-1 and the formation of intracellular conduits and facilitates viral transmission to target cells.

The p12 protein resides in the endoplasmic reticulum, which has a role in protein and lipid synthesis, carbohydrate metabolism, and calcium concentration regulation. Within the ER, p12 is able to mediate an increase in cytosolic calcium in T-cells by increasing calcium release from the ER through inositol trisphosphate receptors and from capacitative calcium entry through Ca^2+^ channels at the plasma membrane in response to the lower ER calcium content (Figure 2(1)) [46,47]. By depleting ER calcium stores and increasing cytosolic calcium, p12 is able to modulate a range of processes including T-cell proliferation, viral replication, and viral spread. Early studies on *orf-I* showed that p12 is able to activate nuclear factor of activated T-cells (NFAT), which is dependent on calcium-binding proteins for its dephosphorylation and nuclear import, to increase T-cell proliferation (Figure 2(2)) [46–48]. Furthermore, p12 can impact other calcium-regulated proteins, including the transcriptional coactivator p300, which can modulate transcription of viral genes from the HTLV-1 LTR [49,50]. Moreover, p12 can promote cell-to-cell viral spread by inducing lymphocyte function-associated antigen 1 (LFA-1) clustering on T-cells through a calcium-dependent mechanism (Figure 2(5)) [51].

#### 2.1.2. NFAT Activation and Signaling

Prior to the discovery of p12 cleavage, expression of *orf-I*-encoded proteins (ORF-I) was shown to enhance T-cell proliferation [46,51,52]. Early studies found that ORF-I was able to mediate activation of NFAT, a transcription factor that regulates activation, proliferation, and differentiation of T-cells [46–48]. In uninfected cells, NFAT can be activated through a complex TCR signaling cascade. Following TCR engagement at the cell surface, the protein tyrosine kinases Lck and Fyn phosphorylate TCRζ and the CD3 subunits. These phosphorylated domains become the docking sites for ZAP70. Activated ZAP70 phosphorylates linker for activation of T-cells (LAT), which then binds and activates phospholipase C-γ-1 (PLCγ1), leading to the production of inositol-1,4,5-trisphosphate and release of Ca^2+^ from ER calcium stores. The increase in cytosolic calcium activates calmodulin and calcineurin, which dephosphorylate NFAT, allowing for NFAT nuclear import. As discussed in Section 2.1.1, by modulating the regulation of cytosolic calcium levels, p12 is able to mediate NFAT activation and does so independent of the proximal TCR signaling molecules, LAT and PLCγ1 (Figure 2(2)) [47]. Interestingly, p12 is able to bind calcineurin and the calcineurin-binding motif of p12 is homologous to the calcineurin-binding motif of NFAT (Figure 2(2)) [48]. Thus, p12 could both enhance and inhibit NFAT activation by competing with NFAT for calcineurin binding. Further studies found that p8, which localizes at the cell surface, is also able to downregulate NFAT activity, but in a LAT-dependent manner (Figure 2(3)) [52].

#### 2.1.3. Proximal T-Cell Signaling and T-Cell Anergy

Recent studies indicate that p8 decreases T-cell activation by inhibiting proximal T-cell receptor signaling [52]. Upon ligation of the TCR to the major histocompatibility complex class II (MHC-II) of an antigen presenting cell, p8 localizes to the immunological synapse where it decreases phosphorylation of LAT, PLCγ1, and Vav by a LAT-dependent mechanism (Figure 2(3)) [23,52]. By dampening TCR signaling, p8 downregulates NFAT activation, a crucial pathway in T-cell activation [47,52]. Furthermore, the induction of T-cell anergy, a state in which T-cells become unresponsive to TCR stimulation, results in decreased Tax activity and HTLV-1 replication [52]. Lastly, since it has been recently shown that p8 transfers to neighboring cells, it is possible that p8-induced T-cell anergy allows a safe transfer of the virus to target cells [9]. These results may underlie the finding that HTLV-1-infected individuals experience some immune deficiency and are susceptible to opportunistic infections [53,54].

#### 2.1.4. IL-2 Receptor Activation and STAT5 Signaling

HTLV-1-infected T-cells proliferate in the absence of IL-2 and this IL-2 independence correlates with constitutive activation of the Janus-associated kinase and signal transducer and activator of transcription (JAK-STAT) pathway, a transcription factor cascade that affects cell proliferation, differentiation, and apoptosis [55]. Early work showed that ORF-I did not have a role in IL-2 independence since it did not affect expression of the interleukin-2 receptor (IL-2R) or IL-2 responsiveness [56]. Also, expression of ORF-I did not affect phosphorylation of JAK-STAT proteins [56]. However, more recent studies have demonstrated that ORF-I binds the β and γ_c_ chains of the immature IL-2R [57]. This interaction stabilizes the IL-2R β and γ_c_ chains in a pre-Golgi compartment and prevents their trafficking to the plasma membrane, leading to a decrease in IL-2R at the cell surface [57]. Specifically, ORF-I binds the 20 amino acid region proximal to amino acid 350 of the IL-2R β chain that is critical for JAK1 and JAK3 recruitment, which occurs after IL-2 signaling [58]. The interaction of ORF-I and IL-2R leads to an increase in STAT5 phosphorylation and DNA binding activity in the absence of IL-2 [58]. This effect is dependent on the presence of the β and γ_c_ chains and JAK3 [58]. By binding the IL-2R, ORF-I decreases the requirement of IL-2 for proliferation in T-cells in the presence of suboptimal antigen stimulation [58].

### 2.2. MHC-I Degradation

HTLV-1 modulates T-cell activation and, in addition, has evolved mechanisms to avoid immune recognition of infected cells. On the cell surface, MHC-I complexes present peptides to TCRs of cytotoxic T-lymphocyte. In the case of virus-infected cells, this interaction leads to recognition of viral peptides and destruction of infected cells. Prevention of MHC-I expression is useful to a number of viruses to maintain the balance between the host and pathogen. Adenovirus E19 protein can retain MHC-I in the ER by interacting with the α1 and α2 regions of class I heavy chains through a dilysine motif [59]. HCMV type I membrane glycoproteins US2 and US11 target MHC-I heavy chains for degradation by the proteasome [60]. Additionally, HIV Nef and Vpu proteins accelerate endocytosis of MHC-I complexes and bind to and destabilize newly synthesized MHC-I, respectively [61–63]. In the ER, p12 binds to newly synthesized MHC-I heavy chains and prevents them from associating with the β_2_-microglobulin, a component of the mature MHC-I complex (Figure 2(4)) [40]. Since improperly assembled proteins are removed from the ER for degradation, the p12-mediated inhibition of MHC-I heavy chain association with the β_2_-microglobulin leads to its degradation by the proteasome and results in decreased MHC-I cell surface expression. By decreasing antigen presentation through degradation of the MHC-I, p12 may diminish presentation of viral peptides and decrease recognition by cytotoxic T-lymphocytes.

### 2.3. Modulation of ICAM

Natural killer (NK) cells recognize and destroy cells that express low levels of MHC-I at the cell surface. ORF-I decreases MHC-I expression to inhibit presentation of viral proteins to cytotoxic T-lymphocytes, which could make HTLV-1-infected cells susceptible to NK cell cytotoxicity [40,64]. In contrast, HTLV-1-infected T-cells are resistant to NK cell-mediated killing [64]. This resistance can be moderately ameliorated by pretreatment of NK cells with IL-2 [64]. Early data demonstrated that several ATL cells lines had altered expression of intercellular cell adhesion molecule 1 (ICAM-1), a glycoprotein that facilitates the interaction between NK cells and T-cells [65]. Furthermore, the majority of HTLV-1-infected primary CD4^+^ T-cells do not express ligands for the NK cell activating receptors, natural cytotoxicity receptors, and NKG2D [64]. Recent work has elucidated these findings as it is now known that ORF-I decreases expression of ICAM-1 and ICAM-2, but not ICAM-3, in T-cells. Thus, ORF-I inhibits NK cell adhesion to T-cells and prevents virus-infected cells from being recognized in the presence of low levels of MHC-I [64].

### 2.4. V-ATPase

The BPV E5 oncoprotein interacts with the 16 kDa subunit of the H^+^ vacuolar ATPase (V-ATPase), resulting in alkalization of the Golgi apparatus [66,67]. The sequence homology between HTLV-1 ORF-I and BPV E5 led to the demonstration that p12 interacts with 16 kDa subunit of the V-ATPase [68,69]. The transmembrane domains of ORF-I appear to be dispensable for binding to the V-ATPase, while conservation of the proline-rich domains between amino acids 36 and 48 contributes to the strength of this interaction [37,69]. The 16 kDa protein is a membrane component of the V-ATPase, which is also found in clathrin coated vesicles, lysosomes, endosomes, Golgi vesicles, endoplasmic reticulum, and synaptic vesicles. This proton pump is responsible for the acidification of these intracellular vesicles [70]. The atypical function of the proton pump through binding of viral proteins such as HTLV-I p12 and BPV E5 proteins may interfere in functions like the dissociation of receptor-ligand complexes and trafficking within the endosomal/lysosomal compartment. In addition, the acidification is essential for the formation of endosome carrier vesicles, which are intermediates between early and late endosomes [71,72]. HTLV-1 is known to infect dendritic cells and the acidification of lysosomes could play an important role in virus entry [7,73,74]. Indeed, the ablation of ORF-I expression impairs HTLV-1 replication in dendritic cells [35].

### 2.5. Modulation of Virus Transmission in vitro and in vivo

HTLV-1 requires *orf-I in vivo* to establish a persistent viral infection [33,35]. Early studies reported that *orf-I* expression was necessary for HTLV-1 infection in the rabbit model [33]. However, these early studies used HTLV-1 clones that, in addition to deleting *orf-I*, produced a frameshift affecting the gene encoding HBZ. Therefore, it is unclear whether these results are due to deletion of *hbz*, *orf-I*, or both. More recently, HTLV-1 molecular clones with nucleotide mutations were used to selectively disrupt *orf-I* expression. This study shows *orf-I* is essential for infectivity in the macaque model but not in the rabbit model [35]. *Orf-I* expression in HTLV-1-infected T-cells enhances virus transmission to target cells [75]. Independent of IL-2, ORF-I increases chemotaxis to facilitate the migration of infected cells toward target cells [75]. Importantly, HTLV-1 infection requires cell-cell contact for efficient transmission through a virological synapse, biofilm-like extracellular viral assemblies*,* or cellular conduits [8–10,76]. Transfer of virus between cells at the virological synapse requires polarization of cytoskeletal proteins and adhesion molecules toward the site of cellular contact [8]. Recent evidence suggests that p8, one of the two *orf-I* products, modulates the clustering of the adhesion molecule LFA-1 to increase the formation of cell-cell contacts and facilitate virus transfer (Figure 2(5)) [9,51]. In addition, p8 promotes the formation of thin membranous cellular conduits, which allows intracellular communication between several cell types [9,77,78]. Through these conduits, the HTLV-1 proteins p8, Gag, and Env are transferred to target T-cells [9]. Altogether, p8 promotes cellular contacts to favor HTLV-1 transmission.

## 3. HTLV-1 p30

**Figure 3.**
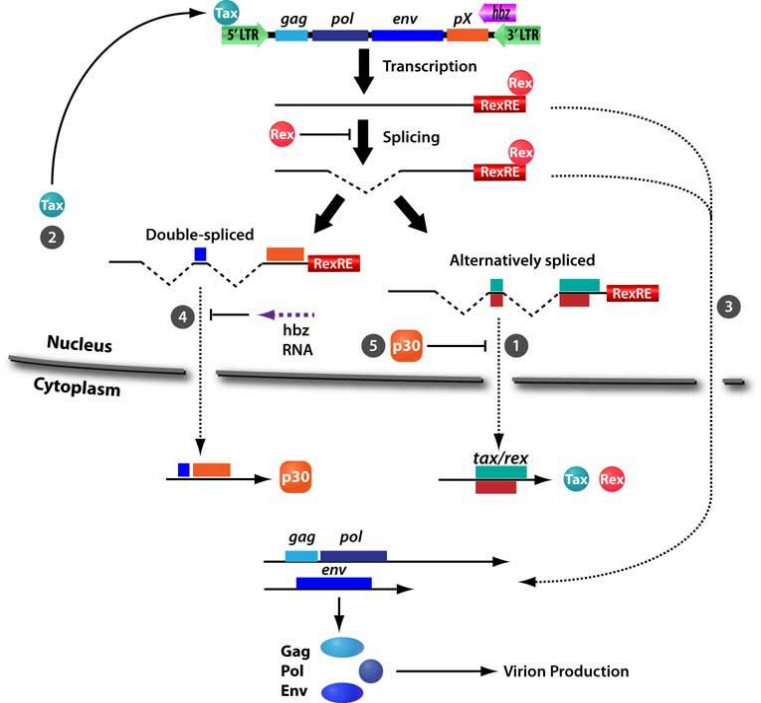
Functions of p30. (**1**) Alternatively double spliced mRNA is translated to form the Tax and Rex regulatory proteins. (**2**) Tax protein localizes to the nucleus to exert its function on the LTR as a positive regulator of viral transcription. (**3**) Within the nucleus, Rex recognizes the Rex-responsive elements (RexRE) of viral mRNA and shuttles these transcripts to the cytoplasm while inhibiting splicing processes. However, some of the viral RNA is processed in the spliced *env* mRNA, the double spliced *p30*, (**4**) and the alternatively spliced *tax/rex* mRNA. *p30* mRNA is subject to negative regulation by *hbz* mRNA. Once p30 protein is produced, it translocates to the nucleus and (**5**) interacts with p30-responsive elements (p30RE) created by the double splicing and therefore is present on *tax/rex* mRNA only. Moreover, p30 interacts with Rex to inhibit Rex-mediated nuclear export of double spliced viral mRNA, including *tax* transcripts. By preventing Tax production, p30 decreases viral transcription.

Initially identified in 1992, the p30 protein is translated from doubly spliced monocistronic mRNA, containing exons 1, 2, and B, transcribed from HTLV-1 *orf-II* (Figure 1) [20,21,79]. p30 is a highly basic protein with a net positive charge that contains three nuclear localization signals (NLS_1_, NLS_2_, and NLS_3_) located between amino acids 66–73, 91–98, and 200–241 and an arginine-rich nucleolar localization/retention (NoRS) domain between amino acids 73–78 [80]. p30 also contains a Rex-binding domain (RexBD) between amino acids 131–164, a p300-binding domain between amino acids 1–132, and a DNA-binding domain between amino acids 100–179 [81]. The DNA-binding domain has been shown to repress LTR-mediated transcription [82]. Notably, HTLV-1 p30 has low genetic variability and is similar to HTLV-2 p28, suggesting a conserved mechanism for negative modulation of virus replication [79,83,84]. p30 shares distant similarities with some human serine-rich transcriptional activators such as Oct-1, Oct-2, Pit-1, and POU-M1 [22]. The p30 protein localizes within the nucleus and nucleolus. p30 shows high mobility within the nucleus, yet it is strongly retained in the nucleolus. Specifically, p30 is located in a granular component where ribosome subunits are assembled and *de novo* mRNA is produced. This localization is consistent with the ability of p30 to bind the ribosomal subunit L18a and to retain in the nucleus the newly transcribed *tax/rex* mRNA (Figure 3(1)) [80]. Since L18a and the eukaryotic initiation factor 3 facilitate re-initiation of translation in the cytoplasm, it is possible that p30 translocates from the nucleoli to the cytoplasm [80]. Similarly, p30 is specifically delocalized from the nucleoli to the nucleoplasm upon DNA damage to interfere with DNA repair processes [85]. In addition, p30 nucleolar retention signal mutants have similar functionality as wildtype p30, which raises questions about the function of nucleolar localization. A recent hypothesis suggests that p30 retention within nucleoli may serve as a reservoir for when the protein is needed in the nucleus [85]. Interestingly, it has recently been shown that *hbz* mRNA can regulate the production of p30, probably acting as an antisense RNA to silence its expression (Figure 3(4)) [86]. Localization of p30 within the nucleus and nucleolus suggest that this protein may mediate critical cellular processes such as cell cycle progression, DNA repair, and mRNA export [87]. Though the main cellular target of HTLV-1 is CD4^+^ T-cells, the virus is able to infect CD8^+^ T-cells, B-cells, macrophages, and dendritic cells. Intriguingly, recent studies have shown that in a macaque model, ablation of p30 within the HTLV-1 provirus severely affects infectivity and leads to reversion of the virus to the wild type genotype [35]. This observation has been confirmed *in vitro* by p30 knockout in HTLV-1-infected human primary and monocyte-derived dendritic cells, in which infection is not sustained over time. In T-cells, p30 is not required *in vitro* for efficient viral replication [35].

### 3.1. Inhibition of Nuclear Export of Tax/Rex mRNA

In contrast to HTLV-1 Tax and Rex, which enhance viral replication, p30 promotes virus latency by retaining *tax/rex* mRNA within the nucleus to prevent its export to the cytoplasm (Figure 3(5)) [88]. By downregulating Tax and Rex production, p30 suppresses viral replication. p30 interacts with the p30 mRNA-responsive element (p30RE) of *tax/rex* mRNA and with Rex at the RexBD [89]. Interestingly, p30RE spans the exon junction created after *env* mRNA is spliced, hence p30 binds spliced *tax*/*rex* mRNA but not the unspliced and singly spliced viral RNA [81]. The interplay between Rex and p30 is a regulatory switch between viral replication and latency. Rex binds with high affinity to the Rex-responsive element (RexRE) at the 3′ end of viral mRNA and, together with CRM1, shuttles unspliced *gag/pol* and singly spliced *env* transcripts to the cytoplasm (Figure 3(3)). Once bound to mRNA, Rex is no longer accessible to binding by p30 and is able to shuttle transcripts to the cytoplasm. However, viral mRNA-bound p30 efficiently interacts with Rex, but *tax/rex* transcripts are still retained in the nucleus (Figure 3(5)) [89]. Nuclear retention of viral mRNA is reversed by an excess of Rex, which displaces p30 from the p30RE. A further spliced version of *tax/rex* has been found in HTLV-infected cells, *p21rex*. During splicing of *p21rex*, the p30RE is removed from *rex* mRNA, which allows the transcript to escape p30-mediated nuclear retention. However, the function of *p21rex* is still unknown [88,90,91]. Thus, by retaining *tax/rex* mRNA, p30 decreases the translation of these two positive regulator of viral replication and promotes latency to escape host immune surveillance and to favor propagation through cell division and clonal expansion of infected cells.

### 3.2 Repression of the CRE Pathway

In addition to its posttranscriptional activity, p30 has been shown to function as either a transcriptional activator or repressor. The ability of p30 to induce transcriptional activation *in vitro* is CBP/p300-dependent [92]. CBP/p300 are known binding partners of CREB and Tax and are required for strong activation of the viral LTR [92]. Several other cellular and viral proteins bind CBP/p300, including members of the Jun-family, c-Myb, c-Fos, STAT1/2, NF-κB, p53, and TATA-binding protein (TBP) [82]. p30 disrupts CREB-Tax-p300 complex formation on the TRE (Tax-responsive element) of the viral LTR, resulting in repression of HTLV-1 transcription [82,93]. p30 has also been reported to differentially regulate transcription from the viral TRE and cellular CREB-responsive elements (CRE) *in vitro* and *in vivo* independent of Tax expression [94,95]. While p30 has been shown to repress CRE-driven gene expression in a dose-dependent manner, low concentrations of p30 enhance LTR activity [95,96]. Therefore, the expression level of p30 may play an important role in its function in the cell. Because p30 suppresses Tax production by retaining *tax/rex* mRNA in the nucleus, it affects CRE- and TRE-mediated transcriptional activation [88]. Interestingly, histone acetyltransferase (HAT) activity of p300 modulates p30-dependent transcriptional downregulation, whereas p30-dependent LTR repression is enhanced by deacetylation and inhibited by acetylation [82,93].

### 3.3. Transcriptional and Posttranscriptional Regulation

Microarray gene expression analyses of human T-cells showed that HTLV-1 p30 affects a number of cellular genes at the transcriptional level. p30 alters expression of a variety of gene families including those that have a role in transcription, translation, cell cycle progression, DNA replication and repair, cell signaling, angiogenesis, cell migration, and apoptosis [97]. Furthermore, p30 is able to retain some cellular transcripts within the nucleus, similarly to viral *tax/rex* mRNA. Included among these transcripts are MDM4, which is a regulator of p53, and HDAC3, which is a histone deacetylases involved in transcriptional repression [97]. Moreover, p30 has been shown to interact with the cellular transcription factor PU.1 in human macrophages. PU.1 is involved in a variety of cellular pathways including signal transduction by Toll-like receptor-4 (TLR-4) [98]. The interaction of p30 and PU.1 leads to inhibition of the DNA binding and transcriptional activity of PU.1. This, together with the p30-mediated inhibition of GSK-3β, yields decreased expression of TLR-4 at the cell surface, resulting in decreased secretion of pro-inflammatory cytokines, such as MCP-1, TNF-α and IL-8, and an increase of the anti-inflammatory cytokine IL-10 [98].

### 3.4. Cell Cycle and DNA Repair

Expression of p30 results in the accumulation of T-cells in the G2 phase of the cell cycle. p30 is able to enhance phosphorylation and activation of check point kinase 1 (Chk1). Chk1 is activated by ATM/ATR kinase following single strand DNA damage and results in a G2 arrest of the cell cycle [99]. p30 specifically binds to ataxia-telangiectasia mutated (ATM) and regulator of 20S proteasome activators γ (REGγ) in multiprotein high molecular weight complexes. By binding to ATM, p30 prolongs cell survival following DNA damage by inhibiting ATM autophosphorylation and subsequent activation of proteins involved in DNA repair, cell cycle check points, and apoptosis [100]. The effect of p30 interactions with REGγ remains to be determined. REGγ promotes formation of the REG-20S proteasome complex and has a critical role in a number of cellular processes, such as cell cycle progression and transcriptional regulation, and can degrade proteins in a ATP- and ubiquitin-independent manner. REGγ is localized in the nucleus where it interacts with and stabilizes p30, thereby altering p30 turnover [100]. In addition, p30 is able to bind to and prevent complex formation of cyclin E and cyclin-dependent kinase 2 (CDK2). Disruption of this complex prevents phosphorylation of retinoblastoma and subsequent E2F-mediated transcription, two key steps for G1/S transition [101]. By inhibiting cyclin E-CDK2 complex formation, p30 delays entry of cells into the S phase of the cell cycle. The inhibition of cell cycle progression is consistent with the observation that dendritic cells isolated from peripheral blood of ATL patients are unable to stimulate proliferation of CD4^+^ and CD8^+^ T-cells [102]. p30 is also able to interact with multiple proteins involved in DNA repair processes [85]. Upon DNA damage, p30 specifically delocalizes from the nucleolus to interact with and affect correct assembly of MRN complexes (Mre11-Rad50-Nbs1). MRN complexes are a key factor of DNA repair and contribute to homologous recombination (HR) during the S phase of the cell cycle. In contrast, during G1 or M phases, DNA damage is preferentially repaired by the nonconservative nonhomologous end joining (NEHJ) pathway. By interacting with these complexes, p30 activates a shift from conservative HR to the error-prone NEHJ pathway, thus favoring an accumulation of genomic alterations. Overall, these observations suggest that p30 could contribute to the accumulation of mutations that are characteristic of transformed HTLV-1-infected T-cells.

### 3.5. Requirement of orf-II in Viral Persistence in Animal Models

Despite the effort of multiple laboratories to define the role of HTLV-1 p30, its exact function and relevance *in vivo* still remains elusive. It has been previously shown *in vitro* that the ablation of p30 expression does not affect viral infectivity of HTLV-1 in human primary cells and that p30 is dispensable for viral replication and immortalization of primary human T-lymphocytes [31,103]. It should be noted that the *in vitro* infectivity of p30-ablated HTLV-1 was not sustained over time in primary dendritic cell [35]. Other observations, such as the presence of antibodies to p30 during infection may provide evidence of the importance of p30 *in vivo* [32,104]. It was first shown that the ablation of p30 expression *in vivo* result is a dramatic decrease of HTLV-1 viral load in a rabbit model [32]. However, the mutation introduced to prematurely stop the translation of *p30* affected *hbz* as well, whose ablation alone decreases viral replication [105]. By inserting an artificial 24 base pair linker containing a premature termination codon in the *p30* ORF, a significant frameshift occurred in the antisense *hbz* ORF [32]. The consequence of this frameshift is unknown, but it most likely affected *hbz* expression. Later studies have used different mutations to ablate *p30* expression while preserving *hbz* [35]. These *in vivo* studies showed that p30 is not necessary for HTLV-1 infectivity in a rabbit model as viral replication was not affected by p30 ablation [35]. In contrast, the mutation of *p30* severely affects virus infectivity in macaques, although a sufficient level of viral replication occurred to allow the reversion to wild type *p30* over time [35].

## 4. HTLV-1 p13

**Figure 4.**
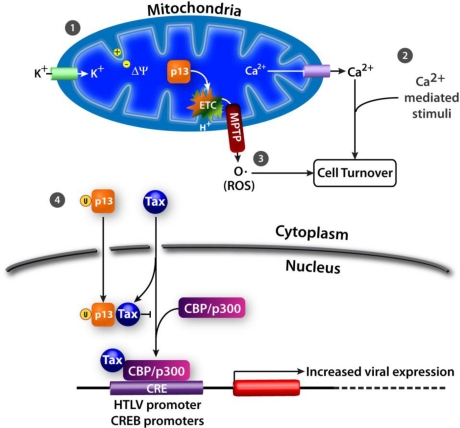
Functions of p13. In mitochondria, p13 mediates (**1**) K^+^ influx, inner mitochondrial membrane potential, and electron transport chain activity to affect (**2**) Ca^2+^ signaling and (**3**) ROS production. (**4**) In the presence of Tax, p13 is ubiquitinated and translocates to the nucleus. In the nucleus, p13 inhibits Tax-CBP/p300 complex formation to decrease transcription of cellular and viral genes.

Differential splicing of mRNA from HTLV-1 *orf-II* results in production of the p13 protein [20–22,106]. p13 is translated from singly spliced monocistronic mRNA to form a highly basic 87 amino acid protein that corresponds to the carboxyl terminus of p30 (Figure 1) [21]. The p13 protein has been predicted to contain a short hydrophobic leader sequence between amino acids 1–5, an amino terminus mitochondrial targeting signal (MTS) in a positively charged amphipathic alpha helix between amino acids 22–31, a transmembrane domain between amino acids 30–40, a flexible hinge region between amino acids 42–48, and a carboxyl terminus β-sheet hairpin structure between amino acids 65–75 that is homologous to an α-bungarotoxin-binding peptide [107–109]. The carboxyl terminus region contains multiple PXXP motifs that may mediate Src homology 3 (SH3) ligand binding. In addition, the carboxyl terminus region contains a cryptic nuclear localization sequence (NLS) [80]. The p13 protein is mainly localized within the inner membrane of mitochondria [107,108]. However, when expressed at high levels, p13 is able to localize within the nucleus and, when coexpressed with Tax, is directed to nuclear speckles [39]. Localization of the p13 protein within mitochondria and the nucleus suggests that this protein may modulate effects on apoptosis and transcriptional regulation (Figure 4).

### 4.1. K^+^ Influx, Inner Mitochondrial Membrane Potential, and Electron Transport Chain Activity

The p13 protein was first shown to induce changes in mitochondrial morphology and distribution [107,108,110]. Mitochondria of p13-expressing cells are clustered and have a rounded ring or crescent-like form that differs from the typical filamentous shape and interconnected mitochondrial network that occurs in normal cells. The rounded shape of mitochondria in p13-expressing cells is formed by osmotic swelling in response to energy-dependent uptake of monovalent cations, such as K^+^ [107,108,110]. p13 mediates this effect by altering the inner mitochondrial membrane potential (Δψ) to change K^+^ permeability of these organelles. The effects of p13 on K^+^ permeability are dose-dependent (Figure 4(1)). At low concentrations, p13 induces mitochondrial swelling without causing mitochondrial depolarization or cytochrome *c* release which is reversed by mitochondrial depolarization using protonophores. Cytochrome *c* is a component of the electron transport chain in mitochondria and is involved in initiation of apoptosis. At higher concentrations, p13 induces irreversible mitochondrial swelling, depolarization, and cytochrome *c* release. The change in p13-mediated mitochondrial morphology is similar to, but distinct from, the changes induced by the mitochondrial permeability transition pore (MPTP), a large nonspecific channel that regulates cytochrome *c* release and apoptosis.

p13-induced K^+^ influx and mitochondrial membrane depolarization stimulates electron transport and mitochondrial respiration, which increases O_2_ consumption [110]. Modulation of respiratory chain activity by p13 is accompanied by increased mitochondrial reactive oxygen species (ROS) production (discussed in Section 4.3) [110,111]. Increased ROS levels, together with mitochondrial membrane depolarization, decrease the opening threshold of the MPTP to promote pro-apoptotic signaling. Interestingly, at low concentrations of p13, increased electron transport chain activity dampens the effects of p13 on Δψ by extruding H^+^ from the matrix [110].

### 4.2. Ca^2+^ Homeostasis

Changes in mitochondrial Δψ also regulate intracellular Ca^2+^ homeostasis (Figure 4(2)) [107,110,112,113]. This effect is tightly linked to the ability of p13 to induce mitochondrial K^+^ influx and depolarization [113]. A p13 peptide was found to induce rapid efflux of Ca^2+^ from preloaded mitochondria [107]. Though p13 reduces mitochondrial Ca^2+^ uptake, it does not significantly affect overall change in cytosolic Ca^2+^ concentration, suggesting that p13-mediated mitochondrial depolarization may alter Ca^2+^ concentration only locally [113]. By altering Ca^2+^ homeostasis, p13 increases the sensitivity of cells to Ca^2+^-mediated stimuli [112]. Increased apoptosis is observed in p13-expressing cells upon treatment with C2 ceramide, which induces influx of Ca^2+^ into mitochondria and opening of the MPTP [112]. Additionally, treatment of cells with histadine results in a rise in cytosolic Ca^2+^ levels, leading to phosphorylation of CREB on serine 133 [112]. When p13 is expressed in cells treated with histadine, there is increased nuclear accumulation of phosphorylated CREB [112]. In cells with mitochondrial defects, such as is observed in p13-expressing cells, increased CREB phosphorylation has been shown to impair cell proliferation [114].

### 4.3. ROS Production

In isolated mitochondria, p13 increases ROS production and this effect is associated with K^+^ influx, mitochondrial membrane depolarization, and activation of the electron transport chain (Figure 4(3)) [110,111]. Unexpectedly, in transformed T-cells cultured using standard conditions, p13 does not increase ROS production [111]. However, in response to glucose deprivation, p13 increases ROS production and cell death in these cells [111]. There is a distinct gradient of ROS accumulation between primary and transformed T-cells, with very low levels observed in resting cells, higher levels in stimulated cells, and substantially higher levels in transformed cells [111]. In contrast to the effects of p13 on transformed T-cells, expression of p13 in unstimulated primary T-cells induces ROS-dependent T-cell activation and proliferation [111]. Thus, by increasing mitochondrial ROS production, p13 mediates activation of primary resting T-cells while promoting cell death in transformed T-cells. By modulating ROS levels in T-cells, it is possible that p13 has a role in lifelong persistence of HTLV-1 in the host by increasing the pool of untransformed infected cells while decreasing the number of transformed cells.

### 4.4. Effects on Apoptosis

Expression of p13 reduces proliferation rates of transformed cells *in vitro* and tumor growth *in vivo.* As discussed in Section 4.1, MPTP-mediated mitochondrial swelling and altered permeability has a key role in inducing apoptosis. Though p13 triggers similar effects as the MPTP, in addition to inducing cristae fragmentation, it does not directly cause apoptosis or cytochrome *c* release [108]. Instead, p13 increases cell sensitivity to pro-apoptotic stimuli such as Fas ligand (FasL), C2 ceramide, and glucose deprivation [111,115]. The effects of p13 on FasL-mediated apoptosis are enhanced by overexpression of Ras and antagonized by inhibiting Ras farnesylation and subsequent activation [115]. Upon FasL stimulation, farnesylated Ras traffics to mitochondria and directly binds Bcl-2 to inhibit the anti-apoptotic effects of Bcl-2 [115]. Treatment of p13-expressing cells with C2 ceramide results in increased influx of Ca^2+^ into mitochondria followed by opening of the MPTP, leading to cell death [111]. During glucose deprivation, p13 promotes apoptosis by increasing ROS production in transformed T-cells [111]. Consistent with the central role of mitochondria in energy production, cation flux, and apoptosis, p13 is able to affect this organelle to influence cell turnover.

### 4.5. Nuclear Effects

In cells expressing Tax, p13 becomes ubiquitinated and is partially localized within the nucleus (Figure 4(4)) [116]. Interestingly, Tax mediates ubiquitination of p13 though this protein contains no lysine residues. Instead, p13 is likely ubiquitinated on serine and threonine residues and this modification increases the stability of the protein. Within the nucleus, ubiquitinated p13 associates with Tax to inhibit its binding to the CBP/p300 transcriptional coactivator [116]. A decrease in Tax-CBP/p300 complex formation results in decreased Tax-mediated viral gene transcription [116]. Thus, intracellular localization of p13 may be an additional regulatory switch between viral replication and latency.

### 4.6. In Vivo Animal Model

CTLs and antibodies that recognize *orf-II* peptides can be detected in HTLV-1-infected individuals, suggesting that p13 may have an important role *in vivo* [104,117]. In an early study to examine the role of p13 alone *in vivo*, a molecular clone of HTLV-1 mutated to selectively ablate p13 failed to establish viral infection in a rabbit model [118]. However, similar to the rabbit model studies discussed in Sections 2.5 and 3.5, it is unknown whether the mutation that ablated p13 also affected expression of HBZ since the amino acid change that ablated the start codon for p13 in this study would also affect the start codon for HBZ [118]. No studies have been completed that examine the role of p13 alone in the macaque model.

## 5. Conclusions

HTLV-1-associated diseases have long periods of clinical latency with infected individuals having life-long persistence of viral-infected T-cell clones. Thus, HTLV-1-infected T-cells must be able to avoid immune recognition. As transcription of the provirus could lead to antigen presentation and immune recognition, it is necessary for HTLV-1 to maintain low levels of virus replication. *In vivo* studies demonstrate that HTLV-1 requires *orf-I* and *orf-II* for viral persistence. Since these proteins play critical roles in T-cell activation, MHC-I trafficking, cytokine expression, and virus replication, they are likely crucial for HTLV-1 maintenance of low level of virus expression *in vivo*. Together, p12 and p8 decrease ICAM-1, ICAM-2, and MHC-I expression at the cell surface, limiting the ability of NK cells and CTL cells to recognize infected cells. In addition, p8 inhibits proximal TCR signaling upon TCR stimulation to prevent T-cell activation. In contrast, within the ER, p12 promotes Ca^2+^ release and NFAT activation in resting cells. Thus, p12 decreases the IL-2 requirement for proliferation in the presence of suboptimal antigen presentation. Altogether, these data suggest that the concerted expression of p12 and p8 modulate T-cell activation and antigen presentation while promoting proliferation of resting cells, therefore providing new target cells for the virus. *Orf-II* protects HTLV-1-infected cells from immune system recognition by dampening the transcriptional effects of Tax. In the nucleus, p30 binds and prevents export of *tax/rex* mRNA to the cytosol for translation and p13, upon ubiquitination, inhibits Tax-CBP/p300 complex formation. This results in a decrease in the transcriptional activity of Tax and subsequent virus replication. In macrophages, p30 favors the production of anti-inflammatory IL-10 while decreasing the expression of pro-inflammatory cytokines by interacting with the transcription factor PU.1. Furthermore, like *orf-I*, p30 and p13 promote proliferation of resting T-cells. p30 inhibits DNA repair mechanisms to favor the growth and persistence of infected cells. p13 modulates the production of ROS and proliferation of resting cells while promoting apoptosis of transformed cells. The understanding of how these proteins simultaneously protect HTLV-1-infected cells from destruction and promote their expansion leads to new questions. Where, when, and at what level these proteins are expressed *in vivo* still must be elucidated. The answers to these questions will indicate whether these proteins may provide new targets for HTLV-1 therapeutics.
